# Assessment of bacterial communities of *Coptotermes gestroi* termite workers attacking Ironwood trees (*Casuarina equisetifolia*) in Guam for the presence of Ironwood tree decline-associated pathogens

**DOI:** 10.3389/fmicb.2024.1454861

**Published:** 2024-12-16

**Authors:** Garima Setia, Junyan Chen, Robert Schlub, Claudia Husseneder

**Affiliations:** ^1^Department of Entomology, Louisiana State University Agricultural Center, Baton Rouge, LA, United States; ^2^University of Guam, Cooperative Extension Service, Guam

**Keywords:** termite, bacterial taxonomy, diversity analysis, metataxonomics, amplicon sequencing, 16S

## Abstract

The Ironwood tree (*Casuarina equisetifolia*) holds a significant ecological role in Guam where a decline in Ironwood trees was first documented in 2002. Studies have linked the Ironwood tree decline (IWTD) to bacteria from the *Ralstonia solanacearum* complex and wetwood bacteria, specifically *Klebsiella oxytoca* and *Klebsiella variicola*. Presence of termites was first found to be associated with IWTD in 2010; however, the role of termites in IWTD is still not clear. The Asian subterranean termite, *Coptotermes gestroi* (Wasmann) (Blattodea: Rhinotermitidae) frequently attacks Ironwood trees. As workers of this soil-dwelling species of the lower termites harbor a diverse microbial community in their bodies, we examined whether *C. gestroi* workers carry IWTD-associated bacteria and could, therefore, act as vectors. We described the bacterial community in *C. gestroi* workers using 16S rRNA gene sequencing and tested the impact of factors related to the location and health of the Ironwood tree the termites were collected from on termite bacterial diversity. Feeding assays were performed to assess if workers show preference in consumption depending on the amount of *Ralstonia* and wetwood bacteria in the food source. Health of Ironwood trees and level of site management impacted the bacterial composition of *C. gestroi* termite workers attacking the trees. Although *C. gestroi* workers equally consumed food sources with high and low *Ralstonia* and wetwood bacteria load in lab experiments, *Ralstonia* and other IWTD-related bacteria were not detected in considerable amounts in termite workers collected from trees. Thus, *C. gestroi* workers are not a vector for bacteria associated with IWTD in Guam.

## 1 Introduction

*Casuarina equisetifolia*, commonly known as Ironwood tree, is an ecologically and economically critical species in Guam, located at the southernmost tip of the Mariana Islands. Ironwood trees display a remarkable tolerance to environmental stressors, including salinity, partial water logging, a spectrum of pests and diseases, and an ability to flourish in nutrient-depleted coral sand (Pinyopusarerk and House, 1993; Elevitch and Wilkinson, 2000). The capacity of the Ironwood tree for soil erosion control and resistance to typhoons further underscores its ecological value. Additionally, the utility of the Ironwood tree extends to applications such as windbreaks, shelterbelts, mulch, and fuelwood (Pinyopusarerk and House, 1993; Schlub, 2013).

Despite the inherent resilience of the Ironwood tree its population in Guam is exhibiting a marked decline that was first documented in 2002 (Mersha et al., 2009, 2010). It was found that the Ironwood trees in Guam are being infected with a disease that leads to gradual thinning of their foliage, progressive dieback of branches, and eventually death of the tree (Mersha et al., 2009, 2010). The dead foliage often remains on the tree giving the tree a singed appearance (Mersha et al., 2009, 2010). In addition to the external symptoms, diseased trees exhibit internal symptoms of wood staining and ooze formation (Schlub, 2013). This condition was called Ironwood Tree Decline (IWTD) (Mersha et al., 2009, 2010; Schlub, 2013).

Various bacteria, including species of *Ralstonia, Klebsiella, Kosakonia, Enterobacter, Pantoea, Erwinia*, and *Citrobacter*, have been identified in the ooze produced by declining Ironwood trees (Ayin et al., 2019). *Ralstonia solanacearum* and certain *Klebsiella* species (*K. oxytoca* and *K. variicola*) are particularly notable, given their frequent isolation from diseased trees and extensive research regarding their association with IWTD (Schlub, 2013; Ayin et al., 2015, 2019). These bacterial species penetrate plant tissues through wounds and migrate upward through the xylem. *Ralstonia solanacearum* is associated with the manifestation of wilt symptoms, while *Klebsiella* species contribute to a brown discoloration of the wood, a condition known as wetwood (Denny, 2006; Hartley et al., 1961). Experimental inoculation of *R. solanacearum* in Ironwood seedlings resulted in wilting and death and *R. solanacearum* has been implicated as a significant predictor of IWTD (Ayin et al., 2019). Wetwood symptoms, however, could not be experimentally induced in healthy Ironwood trees and, therefore, *Klebsiella* species are regarded as potential opportunistic pathogens contributing to IWTD (Ayin et al., 2015, 2019).

The mechanisms facilitating the transmission of putative pathogens linked with IWTD are not yet understood. Numerous biotic and abiotic influences might play a role in the transmission of these pathogens (Agrios, 2008). Insects have to be considered as potential IWTD pathogen vectors due to their well-documented role in the transmission of plant pathogens, including bacteria, viruses, fungi, protozoans, and nematodes (Agrios, 2008; Heck, 2018; Perilla-Henao and Casteel, 2016). Insect vectors can carry pathogens on their mouthparts and transmit them during feeding activities or harbor a pathogen within their bodies where it can multiply as part of its lifecycle before being transmitted to a plant host (Agrios, 2008). Given this context, the role of insect vectors in the transmission of bacteria associated with IWTD presents a valuable line of inquiry.

Termites were identified as one of the insects attacking Ironwood trees in Guam besides beetles and gall wasps (Mersha et al., 2009). A significant association (p < 0.01) was found between the presence of termites and IWTD during a comprehensive survey involving 1,427 trees on the island of Guam (Schlub, 2010, 2013). Signs of termite infestation in Ironwood trees include the presence of tunnels, hollowed tree trunks, and distinctive carton structures in the branches or around the base of the tree (Schlub, 2013). Morphological and molecular characterization techniques, such as DNA barcoding, were used to identify the termite species involved in the attack on Ironwood trees (Park et al., 2019). One of these species attacking Ironwood trees in Guam was identified as *Coptotermes gestroi* (Wasmann), a member of the family Rhinotermitidae (Park et al., 2019).

*Coptotermes gestroi*, a subterranean termite species native to Southeastern Asia, forms colonies under the soil and forages above ground by constructing thin, intricately branched tunnels (Wasmann, 1896; Kirton and Brown, 2003). The worker termites of this species consume a wide variety of materials, including wood, cardboard, paper, leather, rubber, and clothes (Bignell et al., 2011; Kirton and Brown, 2003). Moreover, *C. gestroi* is not selective in its wood consumption as it targets both living and dead trees (Cheng et al., 2014). Worker termites hollow out sections of large trees, while feeding on the wood (Chouvenc et al., 2018; Becker, 1975). Infestation by *C. gestroi* termites has been linked to tree decline and death (Chouvenc et al., 2018).

The species *C. gestroi* is classified within the category of so-called lower termites, a group encompassing the families Archotermopsidae, Mastotermitidae, Stolotermitidae, Kalotermitidae, Hodotermitidae, Stylotermitidae, Rhinotermitidae, and Serritermitidae. The gut microbiota of lower termites harbors a rich community of mutualistic symbionts consisting of cellulolytic flagellates, bacteria and archaea (Brune, 2014; Husseneder, 2023). The termites derive substantial benefits from this symbiotic relationship, as the cellulolytic flagellates aid in wood digestion, while bacteria contribute to the degradation of lignin and chitin, acetogenesis, and nitrogen recycling and fixation, among other vital roles (Brune, 2014; Brune and Friedrich, 2000; Arora et al., 2022; Husseneder, 2023). The microorganisms that are necessary for termite survival are referred to as obligate symbionts and form the core microbiota, i.e., they are present in the workers of most if not all colonies and often in considerable numbers (Cleveland, 1926; Brune, 2014). The majority of core bacteria are typically termite specific and do not occur elsewhere in the environment (Hongoh, 2010; Brune and Dietrich, 2015; Husseneder, 2023).

The gut environment of termites can also accommodate environmental bacteria, provided the chemical composition and pH are conducive to their growth. Worker termites ingest these environmental bacteria during their feeding activities (Keast and Walsh, 1979; Diouf et al., 2018; Fröhlich et al., 2007; Vikram et al., 2021). Some of these ingested bacteria may be plant pathogens that are present on the wood consumed by termites and could potentially be transmitted to a healthy food source during subsequent foraging. Supporting this notion, bacterial genera known to include plant pathogens, such as *Erwinia, Pantoea, Pseudomonas, Burkholderia, Acidovorax, Xanthomonas, Clavibacter, Streptomyces*, and *Ralstonia*, have been detected in the bodies and nests of *Coptotermes* species (Oberpaul et al., 2020). This suggests the potential for IWTD-associated bacteria to be present within the termites that feed on declining Ironwood trees in Guam.

To investigate potential links between IWTD and *C. gestroi* microbiota, we collected termites from Ironwood trees at different stages of IWTD that tested positive or negative for *Ralstonia* and employed 16S rRNA gene amplicon sequencing. Our research objectives were: (1) to conduct taxonomic profiling of bacteria present within whole bodies of *C. gestroi* workers feeding on Ironwood trees in Guam to test for the presence and relative abundance of IWTD-associated pathogens; (2) to assess the potential influence of tree-related and location-related factors on the composition of bacterial communities within *C. gestroi* workers collected from healthy and sick Ironwood trees to understand possible interactions between environmental factors and the microbial ecology within these termite workers; and (3) to explore potential feeding preferences of *C. gestroi* workers in relation to food sources with varied *Ralstonia* and wetwood bacteria content, which would provide insights into whether the termites show a preference or difference in consumption for parts of the trees with lower pathogen content compared to areas with a high pathogen load. This study aims to advance our understanding of the complex interactions between termites, their microbiota, and the health of Ironwood trees, potentially providing strategies to address IWTD in Guam.

## 2 Materials and methods

### 2.1 Termite samples and metadata collection

**Figure 1:**
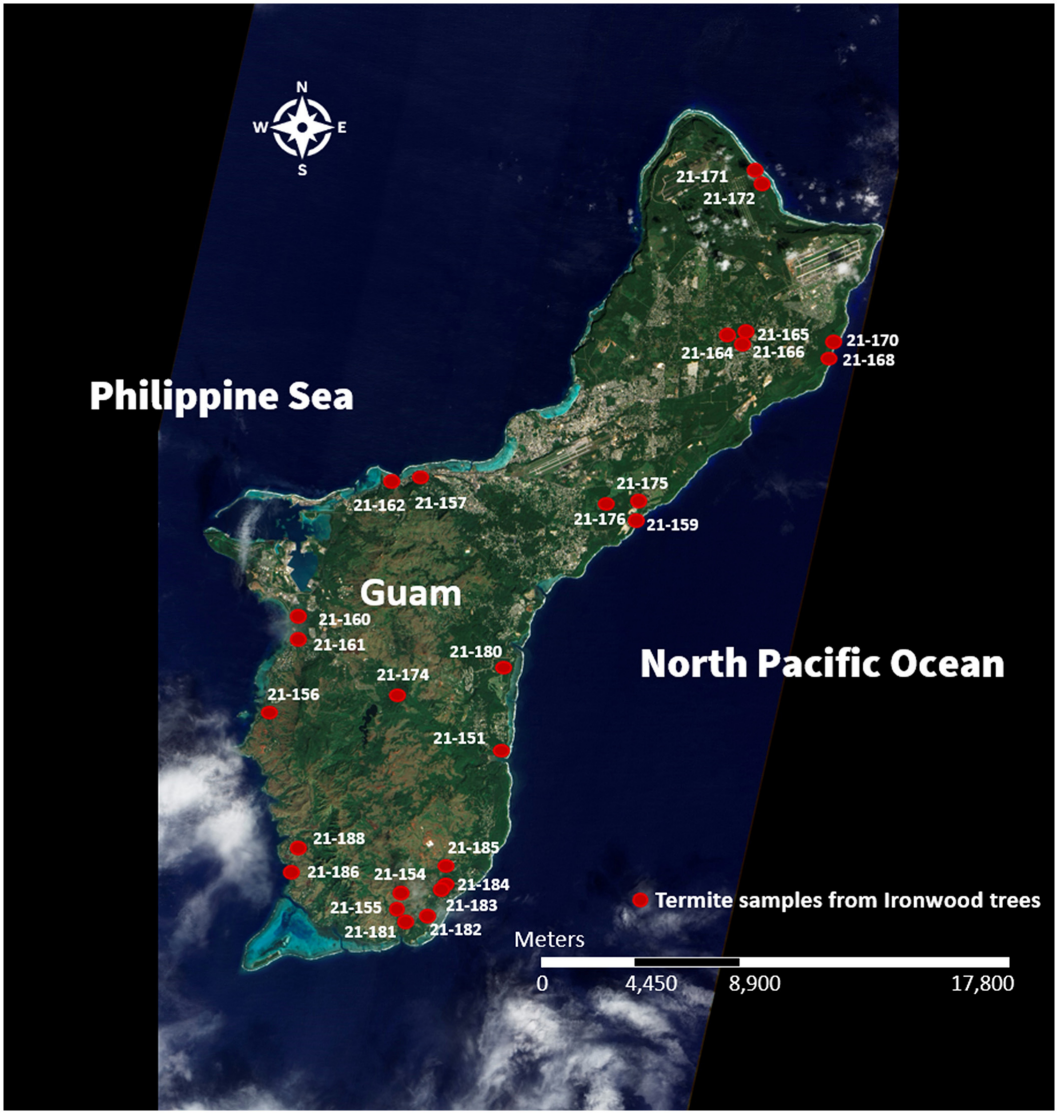
Map of Guam (source: NASA Earth Observatory image created by Jesse Allen and Robert Simmon, using EO-1 ALI data provided courtesy of the NASA EO-1 team and the United States Geological Survey, taken on December 30, 2011 [URL: https://earthobservatory.nasa.gov/images/77189/guam]) showing Ironwood tree sites from where 27 samples of *C. gestroi* termites were collected. Metadata for each termite sample can be found in Supplementary Table S1.

In 2021, the research team from the University of Guam collected 27 *C. gestroi* termite samples from both diseased and healthy Ironwood trees, distributed across 15 distinct locations throughout the island (Figure 1). For each sampling site, at least 21 termites (15 workers and 6 soldiers) were acquired using an aspirator. A third of these specimens were promptly preserved in 70% ethanol (for morphological identification of soldiers and vouchers) and the remaining two-thirds were stored in 95% ethanol (for Illumina sequencing of workers) and shipped to Louisiana State University Agricultural Center. The team from the University of Guam also recorded data regarding tree and location-related factors (Supplementary Table S1, Figure 1) as described in Setia et al., 2023b.

#### 2.1.1 Tree-related factors

Each of the 27 trees from which a termite sample was collected was tested for the Presence of *Ralstonia* (positive vs. negative) using the *R. solanacearum*-specific immunodiagnostic test kit (Agdia, Inc. Indiana. U.S.A.). The test detects *R. solanacearum* through an antigen-antibody reaction without quantifying the concentration. The Decline Severity (DS) of the trees was evaluated based on damage and branch fullness, assigning trees to one of five categories ranging from DS = 0 (symptomless), DS = 1 (slightly damaged), DS = 2 (distinctly damaged), DS = 3 (heavily damaged) and DS=4 (nearly dead) (Schlub, 2013). This evaluation was simplified by reducing the number of DS categories in the factor Tree Health. Symptomless trees (DS = 0) were categorized as healthy while trees showing symptoms of IWTD (DS = 1–4) were jointly categorized as sick (Supplementary Table S1).

#### 2.1.2 Location-related factors

For each of the 27 samples at the 15 sampling locations (Figure 1, Supplementary Table S1), three location-related factors were recorded. Altitude of the tree location was measured in meters above sea level at the tree base and classified as either “low” (≤ 100 m) or “high” (>100 m). Parent Material, the unconsolidated and relatively unweathered source material contributing to soil formation, was categorized into three types: limestone, tuff, and sand based on information from the Natural Resources Conservation Service of the United States Department of Agriculture (https://www.nrcs.usda.gov/). Site Management refers to how extensively the area is maintained by humans, and was classified as not managed, moderately managed, or highly managed.

### 2.2 DNA extraction, primer selection and illumina sequencing

The *C. gestroi* worker samples preserved in 95% ethanol were subjected to DNA extraction using the DNeasy Blood & Tissue kit (Qiagen, Germantown, MA). The total genomic DNA was procured by pooling five workers per sample in lysis buffer and homogenizing them with a sterile pestle (Thermo Fisher Scientific, Wilmington, DE). Quantity of the extracted DNA was measured using an Invitrogen Qubit 4 Fluorometer with the Qubit dsDNA BR Assay Kit (Thermo Fisher Scientific, Wilmington, DE). Twenty microliters (2.5 μl/ng) of the extracted DNA from each termite sample were sent to the University of New Hampshire Hubbard Center for Genome Studies for next-generation sequencing utilizing the 515F and 926R primer pair to amplify the V4 region of the bacterial 16S rRNA genes (Caporaso et al., 2010). The DNA sequencing was performed using the Illumina Nextera Dilute library protocol (Illumina, San Diego, CA). The resultant sequences were deposited in NCBI GenBank as BioProject ID PRJNA883256.

### 2.3 Bioinformatics and statistical analysis

Bioinformatic analysis was performed using the Quantitative Insights Into Microbial Ecology (QIIME2) pipeline, version 2021-4 (Caporaso et al., 2010; Estaki et al., 2020). The Phred quality score of the demultiplexed forward and reverse sequence reads was above 30, thus eliminating the need for truncating. Trimming of primers and adapters, quality filtering, and denoising were performed using DADA2 (Callahan et al., 2016).

Rarefaction curves were generated to assess the adequacy of sequencing depth, sample size, and coverage for capturing the bulk of bacterial diversity within the samples. Sequence depth-based alpha rarefaction curves were generated using QIIME2 by plotting the number of Amplicon Sequence Variants (ASV richness), Faith's Phylogenetic Distance (Faith's PD), and Shannon diversity against the sequencing depth. The sequence reads were subsampled without replacement to a common sequencing depth equivalent to the sample exhibiting the lowest sequencing depth (4,982). Additionally, sample size- and coverage-based alpha rarefaction curves were produced using the R package iNEXT, utilizing Hill numbers (q) equivalent to ASV richness (q = 0), Shannon diversity (q = 1), and Simpson diversity (q = 2) to determine the effective diversity of bacterial communities at the actual sample size of 27 and extrapolate the potential increase in effective diversity if the sample size were doubled (Hsieh et al., 2016).

The ASVs were taxonomically classified with the SILVA 132 reference database (http://www.arb-silva.de) (Quast et al., 2012) using the consensus method in the BLAST algorithm (Camacho et al., 2009) and ASVs with < 97% similarity to reference sequences were filtered out. Sequences were aligned using the MAFFT method and alignments were masked to exclude variable positions (Lane, 1991). Taxa bar plots were generated to represent the relative abundances of taxonomically assigned taxa. The taxonomic assignment of the 20 most frequent ASVs across all samples was confirmed by conducting a BLAST search in NCBI GenBank.

The alpha diversity of bacteria within the termite worker samples was evaluated using four indices: Pielou's evenness, Faith's phylogenetic distance, the number of ASVs (ASV richness), and Shannon diversity (Setia, 2023; Estaki et al., 2020). Group significances for the factors Presence of *Ralstonia*, Tree DS, Tree Health, Location, Parent Material, Site Management, and Altitude, were determined using the Kruskal-Wallis ANOVA (H) test statistic (Kruskal and Wallis, 1952), followed by the Benjamini-Hochberg procedure for false discovery rate correction (Benjamini and Hochberg, 1995). The differentiation of the bacterial composition between termite samples grouped by factors (beta diversity) was analyzed using Permutational Multivariate Analysis of Variance (PERMANOVA) at 999 permutations. The distance metric applied was Weighted Unifrac (Lozupone and Knight, 2005), which takes phylogenetic distances and abundances of bacterial ASVs into account. A PERMDISP test with 1,000 permutations was conducted to account for dispersion and ensure the homogeneity of variance within the sample groups.

### 2.4 Feeding experiments to assess the consumption of *R. solanacearum* by *C. gestroi* workers

#### 2.4.1 Termite collection for feeding tests

Three separate colonies of *C. gestroi* were collected from Apaca Point, Agat (13.40239, 144.66307), Bernard Watson's farm (13.56553, 144.87749), and UOG Ija Experiment Station (13.26523, 144.71623). Plastic milk crate traps (33.2 × 33.2 × 28.1 cm) baited with a lattice structure made from softwood lumber were buried under a soil layer of 3–5 cm as described in Gautam and Henderson (2011). After ~5 months termites were collected by transporting the wood from the crate traps that had been attacked by termites to the laboratory at the University of Guam.

#### 2.4.2 Four-choice test: consumption of *C. equisetifolia* wood with varied bacterial load by *C. gestroi* workers

Wood samples for the four-choice test were obtained from four *C. equisetifolia* trees, each exhibiting either a positive or negative test result for *R. solanacearum* infection, as well as either high or low levels of wetwood bacteria. The tree trunks were cut, cross-sectioned into ~2 cm disks using a circular saw, and small blocks (~0.5 × 2 × 1.5 cm^3^) were chiseled out from the central wetwood region of each disk. All tools were sterilized with 10% Clorox solution after each use. Trees without wetwood stains in their center were assumed to contain low amounts of wetwood bacteria while trees with visible dark stains in the center were assumed to contain high amounts of wetwood bacteria (Setia et al., 2023c). *Ralstonia solanacearum* presence was determined via ImmunoStrip^®^ immunodiagnostic assays (Agdia Inc., Indiana, USA), which utilize antibody-based lateral flow technology to detect specific proteins. The four treatment combinations used in the four-choice test were: (1) *R. solanacearum* negative and low wetwood bacteria (obtained from an Ironwood tree at Yigo Experiment Station, 13°31′59^′′^N 144°51′24^′′^E), (2) *R. solanacearum* positive and low wetwood bacteria (Yigo Experiment Station, 13°31′58^′′^N 144°51′25^′′^E), (3) *R. solanacearum* negative and high wetwood bacteria (Bernard Watson's Farm, 13°20′09^′′^N 144°31′33^′′^E), and (4) *R. solanacearum* positive and high wetwood bacteria (Bernard Watson's Farm, 13°20′10^′′^N 144°31′34^′′^E) (Setia, 2023).

The initial weight of each wood piece was recorded and four wood pieces were spaced out equally in a 145 × 20 mm Petri dish filled with sand at a moisture content of 12% (Setia, 2023). A randomized complete block design involving 15 blocks and five replicates from each of the three termite colonies was used. A total of 300 worker and 30 soldier termites were introduced into each Petri dish. After 3 weeks, the final weights of the wood pieces were measured. The level of consumption was determined by subtracting the final weight from the initial weight of the wood. One-way analysis of variance (ANOVA) followed by a Tukey's Studentized Range test for *post-hoc* analysis, with a significance level of α < 0.05 (SAS 9.4), was used to test for significant changes in the average weight of the wood pieces across different treatments.

#### 2.4.3 Two-choice test: termite consumption of *R. solanacearum* inoculated wood compared to saline control

*Ralstonia* isolate 19-147 (Paudel, 2020) was subcultured from the bacterial ooze of a heavily damaged (DS = 3) Guam Ironwood tree in a shaker incubator with Casamino Acid-Peptone-Glucose (CPG) broth medium (Denny and Hayward, 2001). The optical density of the overnight culture was 2.5 (OD_600_), with 9.251E+9 CFU/ml colony-forming units. Serial dilutions were generated ranging from 10^−1^ to 10^−10^ using 0.85% saline.

Based on a pilot study with *Coptotermes formosanus* Shiraki workers (Setia, 2023), dilutions of 10^−4^, 10^−6^, and 10^−8^ were selected for the tests as these dilutions had shown no adverse impact on the termites. The wood pieces from a healthy *Ralstonia* negative Ironwood tree were oven-dried, weighed, and then inoculated with the bacterial culture at the three dilutions, using 0.85% saline as a control. The inoculated wood pieces were placed in Petri dishes filled with sand at a 12% moisture level. A randomized complete block design with 45 experimental units, combining three colonies, three concentrations, and five replicates, was adopted for this study. A total of 100 *C. gestroi* workers and 10 soldiers were introduced into each Petri dish. After a three-week period, the wood pieces were dried and weighed again, and the consumption data were analyzed using the same methodology as in the four-choice test.

#### 2.4.4 No-choice test: termite consumption of filter paper inoculated with *R. solanacearum* compared to saline control

In the no-choice test, the consumption pattern of termite workers was analyzed by offering the workers filter paper soaked with either 100 μL *Ralstonia* isolate 19-147 at the three different dilutions described above or 0.85% saline. Termites were allowed to feed on the filter papers for 1 or 2 weeks with separate dish sets for each time period. Each of the three termite colonies had five replicate Petri dishes per time period with 50 workers and 5 soldiers per dish. After the end of each feeding period, the dry weight of each filter paper was noted to calculate the consumption. The resultant data was statistically analyzed as described for the four-choice test.

## 3 Results

### 3.1 Number of sequence reads and ASVs

A total of 5,599,951 raw 16S rRNA gene amplicon sequences were obtained from 27 *C. gestroi* samples collected from *R. solanacearum* positive and negative Ironwood trees in Guam. After removing low-quality reads and chimeras, 4,333,087 sequence reads and 8,976 ASVs remained. Removing unassigned ASVs with < 97% similarity to SILVA database references further reduced the dataset to 2,168,762 sequence reads and 747 taxonomically assigned ASVs. The removal of unassigned ASV reduced the minimum sequencing depth across all samples from 16,513 to 4,982 reads, which was used as standardization for rarefaction.

### 3.2 Sequencing depth-, sample-, and coverage-based rarefaction

The sequencing depth-based rarefaction curves of most samples started to level off around 2,000 sequence reads for ASV richness, 8,000 for Faith's PD, and < 2,000 for Shannon diversity (Supplementary Figure S1a), indicating sufficient sequencing depth to capture most bacterial diversity in each sample. The sample-based rarefaction (Supplementary Figure S1b) across the actual 27 samples showed an ASV richness of 747, Shannon diversity of 407, and Simpson inverse of 272. Doubling the sample size by extrapolation increased ASV richness to 1,328, while Shannon and Simpson indices remained approximately at the same level (508 and 289, respectively). Coverage-based rarefaction (Supplementary Figure S1c) showed 90% coverage of bacterial diversity at the achieved sequencing depth. Extrapolating to 95% coverage increased ASV richness to 1,446; however, Shannon diversity only slightly increased to 530 and Simpson indices stayed around 299 with extrapolation suggesting that the increased ASV richness was likely caused by rare ASVs. Overall, rarefaction analyses indicated adequate sequencing and sampling depth to characterize the majority of bacterial diversity within *C. gestroi* samples from Ironwood trees.

### 3.3 Taxa composition

**Figure 2:**
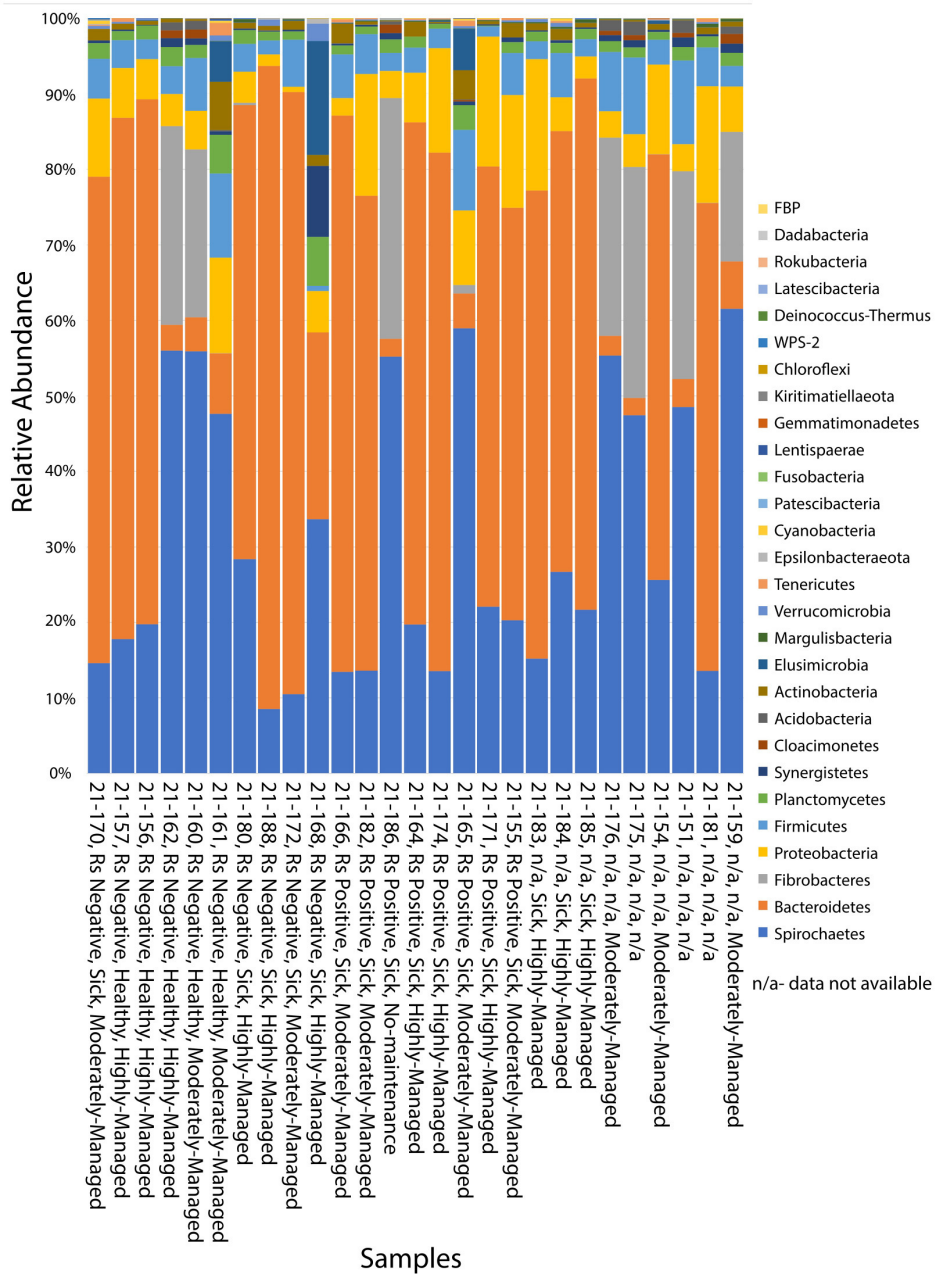
Taxa bar plots showing the relative abundance of bacterial phyla associated with 27 samples of *C. gestroi* workers collected from Ironwood trees in Guam. Phyla are shown in decreasing abundance from bottom to top (Supplementary Table S2). The sample name on the x-axis encodes the following factors: *Ralstonia* (Rs) presence (Positive) or absence (Negative), Tree Health (Healthy or Sick), and level of site management [not managed (None), moderately managed (Moderate), highly managed (High)]. The first number is the sample identifier (Figure 1, Supplementary Table S1). Data that was not available is marked as “n/a”.

A total of 28 bacterial phyla were identified in 27 samples of *C. gestroi* workers gathered from Ironwood trees in Guam (Supplementary Table S2). In order of decreasing abundance, the most prevalent phyla were Spirochaetes (45.46%), Bacteroidetes (23.41%) and Fibrobacteres (15.1%). The minor phyla were Proteobacteria (6.14%), Firmicutes (4.42%), Planctomycetes (1.7%), Synergistetes (0.86%), Cloacimonetes (0.77%), Acidobacteria (0.74%), Actinobacteria (0.72%), Elusimicrobia (0.19%), Margulisbacteria (0.18%) and other phyla with rare representation (0.3%) (Supplementary Table S2, Figure 2).

Only one of the top 20 ASVs in terms of total number of reads was present in all 27 samples; this ASV was an uncultured *Treponema* sp. (Phylum Spirochaetes). Another uncultured *Treponema* sp. was the most dominant ASV, and it was present in 15 out of 27 samples. The second most abundant ASV in terms of read counts was assigned to *Candidatus Azobacteroides* of the Bacteroidetes phylum and was found in 20 samples. An uncultured Spirochaetes bacterium (Phylum: Spirochaetes), another ASV assigned as *Treponema* sp. (Phylum: Spirochaetes), an uncultured delta proteobacterium (Phylum Proteobacteria) and an uncultured Eubacteriaceae bacterium (Phylum Firmicutes) were observed in at least 20 samples. Fourteen among the top 20 ASVs were found in fewer than 20 samples which include the most dominant uncultured *Treponema* sp. mentioned above and another *Ca. Azobacteroides* ASV that was found in one sample (Supplementary Table S3).

Six ASVs that were not in the top 20 ASVs were assigned to taxa that contain pathogenic bacteria species or genera putatively associated with IWTD as these bacteria were identified during the phylogenetic analysis of ooze from Ironwood trees in decline (Supplementary Table S4). However, these ASVs occurred in only a few samples with low abundance and were not exclusive to termites collected from sick trees (Supplementary Table S4). The *Ralstonia* sp. ECPB06, *Enterobacter* sp., *Klebsiella oxytoca*, and *Pantoea* sp. were detected in only one sample with < 10 sequencing reads. *Citrobacter* sp. was also detected in a single sample with 303 reads while *Citrobacter amalonaticus* was observed in 5 samples with a total of 98 sequencing reads (Supplementary Tables S1, S4).

### 3.4 Diversity analysis

**Figure 3:**
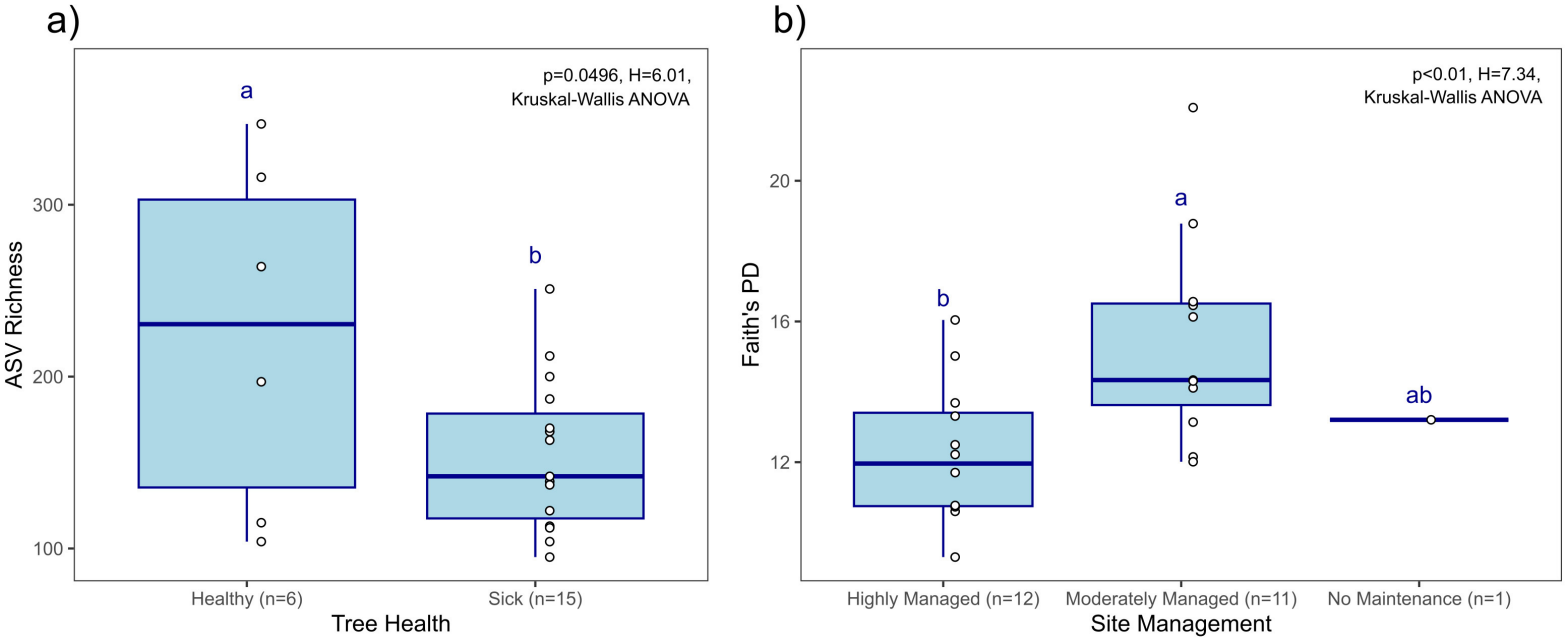
Ironwood tree health and location related factors with significant effects on different aspects of alpha diversity of termite bacteria communities. Different letters indicate significant difference. **(A)** ASV richness of bacteria communities of termites collected from healthy and sick trees. **(B)** Faith's PD of bacterial communities of termites collected from highly, moderately or non-managed sites (Highly Managed, Moderately Managed and No Maintenance).

There was no significant impact of Presence of *Ralstonia*, Tree DS, Altitude, and Parent Material on the alpha diversity (Pielou's evenness, Faith's phylogenetic distance, ASV richness, and Shannon diversity) of the bacterial communities of *C. gestroi* workers collected from Ironwood trees in Guam. However, there were significant differences based on Tree Health and Site Management. Termites collected from healthy trees (n = 6) showed higher bacterial richness (ASV Richness, *p* = 0.0496, H = 6.0075, Kruskal-Wallis ANOVA) compared to those from sick trees (*n* = 15), while termites from moderately managed sites (n = 11) exhibited microbiota with greater phylogenetic distances (Faith's PD, *p* = 0.0067, H = 7.3334) than those from highly managed sites (*n* = 12) (Figure 3). No other alpha diversity metrics were significantly impacted by Tree Health or Site Management.

Beta diversity showed no significant differences in termite bacterial communities based on Presence of *Ralstonia* (*p* = 0.679, pseudo-F = 0.4699), Decline Severity (*p* = 0.463, pseudo-F = 0.8779) and Altitude (*p* = 0.245, pseudo-F = 1.4807, PERMANOVA). Differences were marginal for Tree Health, Site Management and Parent Material (*p* = 0.07, pseudo-F = 2.6804 for each). PERMDISP showed no significant difference in dispersion among the bacterial communities for all the factors (*p* > 0.20, PERMDISP).

### 3.5 Different amounts of *Ralstonia* and wetwood bacteria did not impact feeding behavior of *C. gestroi* workers

Since IWTD pathogens were only detected in small amounts and few *C. gestroi* samples collected from Ironwood trees of Guam, experiments were conducted to investigate the feeding behavior of *C. gestroi* workers in relation to food sources containing pathogens associated with IWTD. The experiments revealed no differences in consumption by termites across multiple pathogen concentrations.

**Figure 4:**
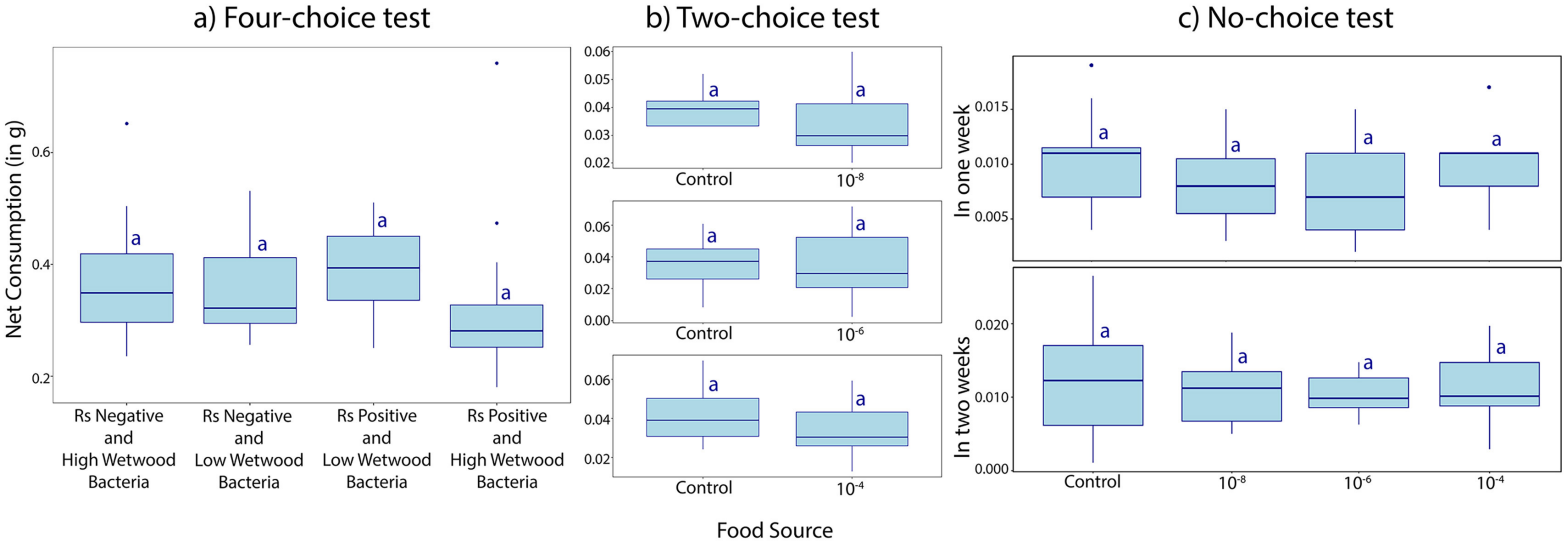
**(A)** Four-choice test measuring net consumption (g) by *C. gestroi* workers of four food sources consisting of natural wood pieces: *R. solanacearum* negative and low amounts of wetwood bacteria, *R. solanacearum* positive and low amounts of wetwood bacteria, *R. solanacearum* negative and high amounts of wetwood bacteria, and *R. solanacearum* positive and high amounts of wetwood bacteria. **(B)** Two-choice test measuring net consumption (g) of wood pieces inoculated with 10^−4^, 10^−6^ and 10^−8^ *Ralstonia* dilution by *C. gestroi* workers in comparison to a control with no *Ralstonia*. **(C)** No-choice test measuring net consumption (g) of filter papers inoculated with 10^−4^, 10^−6^, and 10^−8^ bacterial concentration by *C. gestroi* workers at 1 and 2 weeks. Same letters indicate lack of significant differences determined by One-way ANOVA and Tukey's Studentized Range (HSD) Test.

In the four-choice tests, where termites were fed with four different natural wood sources from *Ralstonia* positive or negative Ironwood trees with high or low amounts of wetwood bacteria, termites showed no discriminatory feeding behavior between the four treatment combinations. No significant differences in the net consumption were observed among the wood from *Ralstonia-*positive or negative Ironwood trees with high or low amounts of wetwood bacteria (p = 0.418, One-Way ANOVA followed by HSD test) (Figure 4A). Termite feeding activity was robust across all replicates regardless of the wood's pathogen status.

In two-choice tests that compared consumption of Ironwood pieces inoculated with three different dilutions of *Ralstonia* (10^−4^,10^−6^, and 10^−8^) against wood that is not inoculated with *Ralstonia*, termites displayed consistent feeding patterns across all three dilutions tested. The consumption of inoculated wood did not differ significantly from the consumption of wood without *Ralstonia* (*p* = 0.141, *p* = 0.823, *p* = 0.251 for the three concentrations, respectively, *n* = 15, One-Way ANOVA followed by HSD test, Figure 4B).

In no-choice tests no significant difference was observed between consumption of filter paper inoculated with *R. solanacearum* at different concentrations (10^−4^, 10^−6^, 10^−8^) and the control, both, after feeding for 1 week (*p* = 0.249) and 2 weeks (*p* = 0.876, One-Way ANOVA followed by HSD test, Figure 4C). The average mortality rate (15.3 ± 8.12%) was constant among the treatments. The results of the three feeding experiments indicate that *C. gestroi* workers do not avoid food sources containing *Ralstonia* or wetwood bacteria, regardless of pathogen concentration or exposure duration.

## 4 Discussion

The bacterial composition of freshly collected whole bodies of *C. gestroi* workers was described with 16S rRNA gene sequencing to investigate the possibility that *C. gestroi* workers could play a role as pathogen vectors for IWTD in Guam. Spirochaetes (45.46% relative abundance), Bacteroidetes (23.41%), Fibrobacteres (15.1%), Proteobacteria (6.14%), and Firmicutes (4.42%) were found to be the dominant phyla in *C. gestroi* workers from Guam. All of these dominant phyla except the Fibrobacteres were also found in two previous deep sequencing studies in *C. gestroi* workers (Do et al., 2014; Oberpaul et al., 2020) and are, therefore, core phyla. However, the relative ranking according to the abundance of each phylum differed among these studies, which might be due to geographical population differences and/or differences in sample collection, rearing and sequenced 16S region. Do et al. (2014) performed metagenomic sequencing and found Firmicutes (22.48%), Proteobacteria (17.84%), Spirochaetes (17.40%), Bacteroidetes (11.60%) and Synergistetes (4.27%) to be the most dominant phyla. In contrast to our study, they used lab-reared, pinewood-fed *C. gestroi* workers from Vietnam. In addition, they targeted free-living gut microbiota and did not fully capture bacteria that are closely associated with gut protozoa; thus, their approach resulted in a significant underrepresentation of the phyla Spirochaetes and Bacteroidetes which are primarily endosymbiont bacteria of protozoa and constitute more than 70% of bacteria in the hindgut of *Coptotermes* species (Noda et al., 2005), and bacteria attached to the gut walls, accounting for 3–20% of hindgut bacteria of *Coptotermes* species (Nakajima et al., 2006). Instead of Spirochetes followed in abundance by Bacteroidetes and Fibrobacteres as our study found, Oberpaul et al. (2020) reported Bacteroidetes (52%), Alphaproteobacteria (12%), Spirochetes (11%), Firmicutes (7%), and Actinobacteria (6%) as the dominant phyla in descending order across three *Coptotermes* species, including *C. gestroi*, with Fibrobacteres representing only a minor fraction of the bacterial community. Similar to our study, Oberpaul et al. (2020) used whole bodies of workers; however, they employed different primers than our study to sequence the V3-V4 region of the bacterial 16S rRNA gene and primer bias is known to cause differences among bacterial communities (Thijs et al., 2017; Haro et al., 2021). Moreover, the termites in Oberpaul et al.'s study were reared for decades in lab colonies fed on birchwood, which also might explain the differences in phyla rankings compared to our study with termites collected from their natural environment.

The high abundance of Fibrobacteres (15.1%) set our study apart from the previous *C. gestroi* bacteria inventories (< 1% in Oberpaul et al., 2020 and no mention in Do et al., 2014). While this discrepancy might have been caused by the differences between the studies mentioned above, horizontal transfer from other termite species coexisting on the same tree might also contribute to the unusual high abundance of Fibrobacteres in *C. gestroi*. Fibrobacteres are usually rare in lower termites like the genus Coptotermes which have cellulolytic flagellates (Brune, 2014). However, Fibrobacteres is typically a dominant phylum in higher termites of the family Termitidae as bacteria of this phylum are important contributors to lignocellulose digestion in termitids that lack cellulolytic flagellates (Brune, 2014; Husseneder, 2023). At least two genera of the Termitidae, Nasutitermes and Microcerotermes, are infesting Ironwood trees in Guam (Park et al., 2019). A termitid from the *Nasutitermes takasagoensis* complex is the termite species most often found to infest Ironwood trees in Guam (Park et al., 2019) and *N. takasagoensis* colonies have been observed to nest and forage in close proximity on the same tree as *C. gestroi* colonies (Park et al., 2019, G. Setia personal observation). As a member of the Termitidae *N. takasagoensis* harbors on average 41% Fibrobacteres in all samples from Guam's Ironwood trees making it the second most abundant phylum (Setia et al., 2023b). *Microcerotermes crassus* samples also contain Fibrobacteres with an average abundance of 28% among their core bacteria (Setia et al., 2023a). In contrast, only half the *C. gestroi* samples contained Fibrobacteres indicating that this phylum is not part of the core microbiota of *C. gestroi* workers but may have been secondarily acquired by some colonies. Interspecific horizontal transfer of microbiota can occur among termite species, either through shared feeding substrates or aggressive encounters (Bourguignon et al., 2014). Although we have no direct evidence of horizontal transfer of bacteria in this study, it is possible that *C. gestroi* workers acquired Fibrobacteres through interaction with *Nasutitermes* or *Microcerotermes* species. Given their symbiotic compatibility with termites, Fibrobacteres might bolster the lignocellulose digestion capacity of *C. gestroi*, particularly under conditions of diminished flagellate populations (Brune, 2014).

The 20 most frequent bacteria ASVs across all *C. gestroi* samples were predominantly uncultured species. Among the most dominant ASVs in terms of relative abundance and ubiquity that were taxonomically assigned to the genus or species level were *Treponema* sp., *Candidatus* Azobacteroides, *Trabulsiella termitis*, and *Wolbachia*, which were found in 15, 20, 13, and 17 of the samples, respectively (Supplementary Table S3). *Treponema* represents a phylogenetically diverse bacterial group within the phylum Spirochaetes (Brune et al., 2022; Husseneder, 2023). *Treponema* spirochaetes can be found in every termite species (Arora et al., 2022). *Treponema* species are involved in lignocellulose hydrolysis, fermentation, acetogenesis, and nitrogen fixation processes within termite guts (Leadbetter and Breznak, 1996; Breznak, 2000; Graber and Breznak, 2004). The presence of *Treponema* in *C. gestroi* aligns with previous studies that have reported their dominance in the gut microbiomes of other lower termites (Hongoh et al., 2003; Köhler et al., 2012). *Candidatus* Azobacteroides, a member of the phylum Bacteroidetes and order Bacteroidales, serves as an intracellular endosymbiont for cellulase-digesting protozoa of the genus *Pseudotrichonympha*, and shares a coevolutionary relationship with its host (Noda et al., 2005; Chen et al., 2022). *Candidatus* Azobacteroides plays a crucial role in nitrogen and carbohydrate metabolism in the rhinotermitid genera *Coptotermes* and *Heterotermes* (Hongoh and Ohkuma, 2018; Arora et al., 2022). This bacterial endosymbiont was first sequenced in a *Pseudotrichonympha* species from *C. formosanus* (Hongoh and Ohkuma, 2018), and related strains were also detected in *C. gestroi* workers (Oberpaul et al., 2020; Chen et al., 2022). The bacterium *Trabulsiella odontotermitis*, belongs to the phylum Proteobacteria and the order Enterobacteriales. *Trabulsiella odontotermitis* is typically associated with various fungus-growing termites and was first isolated from *Odontotermes formosanus*, a fungus-growing termite species (Sapountzis et al., 2015; Chou et al., 2007). *Trabulsiella odontotermitis* is considered as a facultative symbiont and is potentially responsible for carbohydrate metabolism and aflatoxin degradation (Sapountzis et al., 2015). *Trabulsiella odontotermitis* was cultured from *C. formosanus* and used in paratransgenesis-based termite control studies as a genetically engineered termite specific symbiont (Tikhe et al., 2016). *Wolbachia*, an ASV of the phylum Proteobacteria and order Rickettsiales ranked 12th in abundance in our study and found in 17 samples. *Wolbachia* ranks among the most prevalent endoparasites in various insects and nematodes (Taylor et al., 2018; Duron et al., 2008). The *Wolbachia* ASV identified in our study exhibited 100% sequence identity to a *Wolbachia* sequenced from a *Coptotermes lacteus* specimen (NCBI accession number DQ837199.1, Supplementary Table S3). Typically, *Wolbachia* strains infecting lower termites belong to supergroup H, and those infecting higher termites to supergroup F; however, *C. lacteus* Wolbachia were classified into subgroup F (Lo and Evans, 2007). Other lower termite species that harbor supergroup F, include *Coptotermes lacteus, Coptotermes acinaciformis* and *Coptotermes heimii* (Salunke et al., 2010).

Certain bacterial genera previously identified in Ironwood trees affected by IWTD, i. e., *Ralstonia, Enterobacter, Klebsiella, Pantoea*, and *Citrobacter* (Ayin et al., 2019), were found in a few *C. gestroi* worker samples collected from Ironwood trees in Guam but they represented rare ASVs (< 0.02% reads) and were found in < 40% of the samples (Supplementary Table S4). However, of all these bacteria, *R. solanacearum* is the only pathogen associated with IWTD that has been confirmed to cause symptoms in Ironwood trees through isolation and reinoculation. *Ralstonia solanacearum* isolated from ooze of sick Ironwood trees in Guam was able to cause wilting symptoms on healthy Ironwood trees (Paudel, 2020). The isolates of *Klebsiella* sp. from Ironwood trees in Guam were unable to elicit symptoms of wetwood in healthy trees and the remaining bacteria have yet to be tested for their pathogenicity in Ironwood trees (Ayin et al., 2015, 2019).

*Ralstonia* sp. was found in only one of the *C. gestroi* samples which was collected from a severely affected *Ralstonia*-positive tree (Highly damaged DS = 3). However, out of over 2 million sequencing reads across all samples, only nine reads were mapped to *Ralstonia*. This indicates an extremely low likelihood of *Ralstonia* presence in *C. gestroi* worker termite samples, i.e., < 1 in 241,084 bacteria. The failure to detect *Ralstonia* in the majority of samples is unlikely to be due to insufficient sequencing or sampling effort. As confirmed through alpha rarefaction analysis, the sequencing depth, number of samples, and coverage used in this study were adequate to capture most of the bacterial diversity. Therefore, we tested whether the low detection of *Ralstonia* may be due to *C. gestroi* workers avoiding the consumption of pathogen-contaminated food sources. However, in choice tests conducted using wood from healthy Ironwood trees and wood from Ironwood trees with high loads of *Ralstonia* and wetwood bacteria, as well as wood inoculated only with *Ralstonia, C. gestroi* workers showed no preference between healthy wood and wood containing high pathogen loads. In addition, the *C. gestroi* workers consumed the food sources used in no-choice tests evenly irrespective of the bacterial load. This observation suggests that *C. gestroi* workers do not avoid pathogen-contaminated wood, at least within the tested concentration ranges (10^−4^, 10^−6^, 10^−8^ dilutions). In contrast to *C. gestroi* workers in this study, we previously showed that *C. formosanus* workers had reduced consumption at higher concentration of *Ralstonia* (10^−2^ dilution) than employed in the present study (Setia, 2023). Similarly, workers from the major species attacking Ironwood trees, i.e., *N. takasagoensis*, showed a preference for consumption of healthy wood even at low pathogen loads (Setia et al., 2023b). The lack of deterrence caused by bacterial contamination of *C. gestroi* compared to *N. takasagoensis* workers could be attributed to different feeding behaviors of these termite species. *Coptotermes* species are known for their voracious and opportunistic feeding habits, as they can consume anything containing cellulose, as evidenced by the great damage they cause to structures and trees (Cowie et al., 1989). Microbiome analysis and feeding tests suggest that *C. gestroi* workers likely consume *Ralstonia*, given that (1) *Ralstonia* was identified in worker bodies, albeit rarely in terms of sequence and sample numbers, and (2) feeding tests revealed that *Ralstonia* did not serve as a feeding deterrent. However, *Ralstonia* did not establish itself in large quantities in termite bodies, likely due to various reasons. Termites employ both individual and social immunity against foreign bacterial invasions (Rosengaus et al., 1999; Traniello et al., 2002; Cremer et al., 2018), and hygienic behaviors such as grooming, avoidance of infected nest mates, burial or cannibalism of dead termites, prevent most environmental bacteria from colonizing the termite body (Yanagawa and Shimizu, 2007; Rosengaus et al., 1999; Liu et al., 2015; He et al., 2018). The inherent microbiota and the antimicrobial compounds in both the termites' bodies and nests also help prevent the invasion and subsequent colonization by foreign bacteria (Veivers et al., 1982; Dillon and Dillon, 2004; Chouvenc et al., 2013; Soukup et al., 2021; Witasari et al., 2022). Furthermore, the slightly acidic to neutral pH in the gut of *C. gestroi* workers is outside the ideal pH range of 4.5–5.5 for *R. solanacearum* growth (Li et al., 2017).

Although IWTD-associated bacteria were not found in meaningful numbers in *C. gestroi* workers attacking Ironwood trees, and the workers' alpha and beta bacterial diversity was not different whether they fed on *Ralstonia* positive or negative Ironwood trees, consuming diseased Ironwood trees appears to alter certain aspects of bacterial diversity in *C. gestroi* workers. Termites feeding on sick Ironwood trees that showed symptoms of IWTD exhibited a decrease in bacterial richness (alpha diversity) compared to those feeding on healthy trees. The decreased bacterial richness in *C. gestroi* workers collected from sick trees was most likely due to a reduction of closely related bacterial ASVs, since phylogenetic diversity did not differ in regard to Tree Health. These findings are different from a previous study in *N. takasagoensis* workers, which showed similar richness but higher phylogenetic diversity when feeding on sick Ironwood trees compared to samples from healthy trees (Setia et al., 2023b). Tree Health was also marginally associated with differentiation between bacterial communities (beta diversity) of *C. gestroi* workers collected from healthy and sick trees. Previous studies on bleeding canker disease caused by *Pseudomonas syringae* pv. aesculin, showed less microbiome alpha diversity in symptomatic compared to asymptomatic trees (Koskella et al., 2017). The authors claimed that low microbiome diversity, induced by biotic or abiotic stressors, could undermine a plant's defenses, allowing pathogens to infiltrate the plant tissue (Pearce, 1996; Koskella et al., 2017). As the presence of tree disease appears to influence the microbiomes of both the tree (Koskella et al., 2017) and the termites feeding on it (this study and Setia et al., 2023b), there may be a link between the microbiomes of trees and termites that needs further exploration.

The extent of human management of the site where the Ironwood tree is situated impacts IWTD severity (Schlub, 2010). Trees in areas with high management are more likely to experience IWTD than those in less-managed areas (Schlub, 2010). High site management level was associated with a decrease in phylogenetic diversity in the microbiomes of *C. gestroi* (this study) and *N. takasagoensis* (Setia et al., 2023b) workers feeding on trees at intensely compared to moderately managed sites. Site management also was marginally linked to bacterial community differentiation. One explanation could be that highly managed sites have less plant diversity due to removal of unwanted species, and also are exposed to fertilization and weed killers, which in turn might reduce microbial diversity in the soil and consequently in the trees (Lavelle et al., 1995; Wardle et al., 2004). A decrease in microbiota diversity in the soil that subterranean termites like *C. gestroi* workers tunnel through and of the wood they feed on could cause the observed reduction in bacterial phylogenetic diversity of the termites. However, a recent study did not detect an impact of site management on the microbiota of soil collected around Ironwood trees in Guam (Jin et al., 2024). Therefore, further research is required to understand the interplay between site management and the microbiomes of Ironwood trees and termites, and their combined impact on IWTD.

In conclusion, this study suggests that *C. gestroi* workers are not likely to transmit pathogens associated with IWTD in Guam, given their low and infrequent pathogen load. However, the damage caused by termite infestation could provide entry points for plant pathogens, potentially accelerating the disease progression (Agrios, 2008). Alternatively, the observed association between termites and IWTD (Schlub, 2010) might not be due to a causal role of termites in the tree's decline, but rather because the termites opportunistically feed on weakened or dead trees (Sugimoto et al., 2000).

## Data Availability

The datasets presented in this study can be found in online repositories. The names of the repository/repositories and accession number(s) can be found below: https://www.ncbi.nlm.nih.gov/, PRJNA883256.

## Supplementary material

Supplementary Table S1 Location- and tree-related metadata recorded for *C. gestroi* samples. This data was recorded by the staff members from the University of Guam. Data that was not available is marked as “n/a”. A detailed description of each metadata category can be found in Setia (2023).

Supplementary Table S2 Number of rarefied sequence reads and relative abundance of the 28 phyla found across 27 *C. gestroi* samples and the number of samples each phylum was observed in.

Supplementary Table S3 The most abundant 20 ASVs associated with *C. gestroi* samples according to the total number of reads with their assignments in SILVA and NCBI GenBank, their total number of reads along with the number of samples the ASVs were observed in and the average number of reads and standard deviation per sample.

Supplementary Table S4 Six ASVs from *C. gestroi* workers collected from Ironwood trees in Guam were assigned to putative plant pathogenic bacteria species or belonged to genera with known plant pathogens.

Supplementary Figure S1 **(a)** Sequencing depth-based rarefaction curves of bacteria diversity showing the number of ASVs, Faith's phylogenetic distance and Shannon diversity indices in 27 samples of *C. gestroi* workers plotted against sequencing depth. **(b)** Sample-based rarefaction curves with effective bacterial diversity for different metrics plotted against the number of samples. **(c)** Coverage-based rarefaction curves with effective diversity plotted against estimated sample coverage. Solid lines indicate intrapolation up to the actual sample size; dashed lines represent extrapolation to twice the sample size. Rarefaction was performed over the total bacteria diversity (with and without taxonomical assignment).
