# Supplementary figures and images for: Assessment of bacterial communities of Coptotermes gestroi termite workers attacking Ironwood trees (Casuarina equisetifolia) in Guam for the presence of Ironwood tree decline-associated pathogens
Source: Front Microbiol. 2024 Dec 16;15:1454861. doi: 10.3389/fmicb.2024.1454861 (PMC11689660; doi:10.3389/fmicb.2024.1454861)

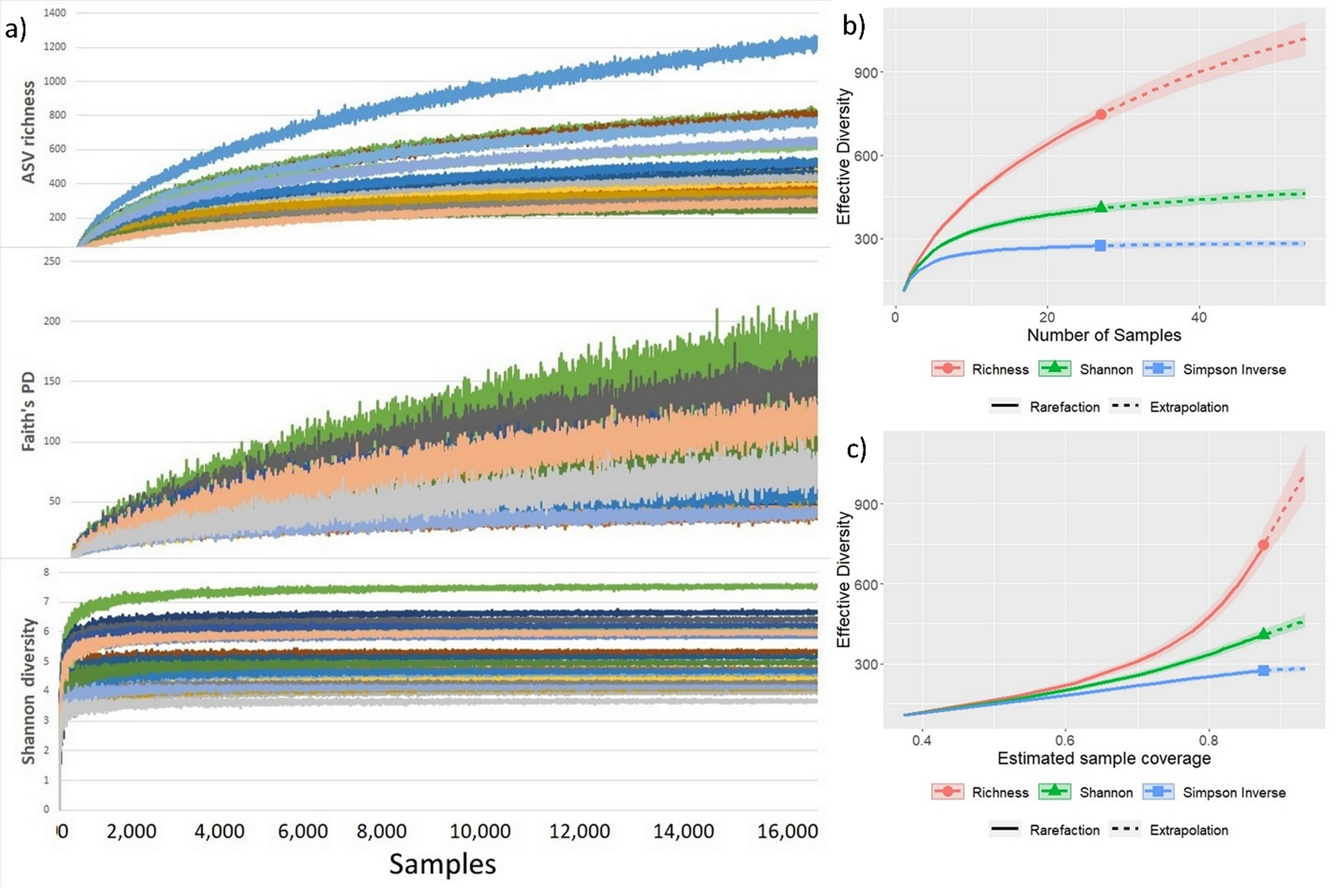

Supplement: Supplementary Figure S1 — (a) Sequencing depth-based rarefaction curves of bacteria diversity showing the number of ASVs, Faith's phylogenetic distance and Shannon diversity indices in 27 samples of C. gestroi workers plotted against sequencing depth. (b) Sample-based rarefaction curves with effective bacterial diversity for different metrics plotted against the number of samples. (c) Coverage-based rarefaction curves with effective diversity plotted against estimated sample coverage. Solid lines indicate intrapolation up to the actual sample size; dashed lines represent extrapolation to twice the sample size. Rarefaction was performed over the total bacteria diversity (with and without taxonomical assignment). [file Image_1.tiff]
